# A Novel Iterative Scheme and Its Application to Differential Equations

**DOI:** 10.1155/2014/605376

**Published:** 2014-03-16

**Authors:** Yasir Khan, F. Naeem, Zdeněk Šmarda

**Affiliations:** ^1^Department of Mathematics, Zhejiang University, Hangzhou 310027, China; ^2^Modern Textile Institute, Donghua University, Shanghai 200051, China; ^3^Department of Mathematics, Faculty of Electrical Engineering and Communication, Brno University of Technology, Technicka 8, 61600 Brno, Czech Republic

## Abstract

The purpose of this paper is to employ an alternative approach to reconstruct the standard variational iteration algorithm II proposed by He, including Lagrange multiplier, and to give a simpler formulation of Adomian decomposition and modified Adomian decomposition method in terms of newly proposed variational iteration method-II (VIM). Through careful investigation of the earlier variational iteration algorithm and Adomian decomposition method, we find unnecessary calculations for Lagrange multiplier and also repeated calculations involved in each iteration, respectively. Several examples are given to verify the reliability and efficiency of the method.

## 1. Introduction

Over the last few decades several analytical/approximate methods have been developed to solve nonlinear ordinary and partial differential equations. For initial and boundary-value problems in ordinary and partial differential equations, some of these techniques include the perturbation method [1], the variational iteration method [2–4], the decomposition method [5–8], and the homotopy methods [9–11].

The Adomian decomposition method [12–16] for solving differential and integral equations, linear or nonlinear, has been the subject of extensive analytical and numerical studies. The method, well addressed in [12–16], has a significant advantage in which it provides the solution in a rapid convergent series with elegantly computable components. In recent years, a large amount of literature has been developed concerning the application of Adomian decomposition method in applied sciences. In addition, the method reveals the analytical structure of the solution which is absent in numerical solutions.

He's variational iteration method [2–4] is based on a Lagrange multiplier technique developed by Inokuti et al. [17]. This method is, in fact, a modification of the general Lagrange multiplier method into an iteration method, which is called correction functional. The method has been shown to solve effectively, easily, and accurately a large class of nonlinear problems [18–23]. Generally, one or two iterations lead to high accurate solutions.

In the present study, we have linked up variational iteration method and Adomian decomposition method through Lagrange multiplier, which shows that VIM is another form of expressing ADM and vice versa. This study reveals that there is no need to integrate the differential equation again and again as we do in Adomian decomposition method. Advantage of new iterative scheme over the variational iteration method is that it avoids the unnecessary calculations and we can construct Lagrange multiplier very easily without construction of the correctional functional.

## 2. New Formulation for Adomian Decomposition Method and Variational Iteration Algorithm II

In order to elucidate the solution procedure, we consider the following *n*th order partial differential equation:

(1) $$\begin{array}{c} L^{n}f\left(x,t\right)=Rf\left(x,t\right)+Nf\left(x,t\right)+g\left(x,t\right),\;t>0,\;\;x\in L, \end{array}$$

where *L* ^*n*^ = ∂^*n*^/∂*t* ^*n*^, *n* ≥ 1, *R* is a linear differential operator, *N* is a nonlinear differential operator, *R* and *N* are free of partial derivative with respect to variable *t*, and *g* is the source term. As we are familiar with the fact that in all kinds of iteration techniques, except the operator rest of the terms, are treated as a known function on the behalf of initial guess. In this present newly proposed idea, we have used the same concept. We have bound all terms in one function except operator. Consider

(2) $$\begin{array}{c} g+Nf+Rf=F\left(t,x,g,f,\frac{\partial f}{\partial x},\frac{\partial^{2}f}{\partial x^{2}},\ldots \right). \end{array}$$

By incorporating (2) in (1), we get

(3) $$\begin{array}{c} L^{n}f=F\left(t,x,g,f,\frac{\partial f}{\partial x},\frac{\partial^{2}f}{\partial x^{2}},\ldots \right). \end{array}$$

On integrating (3), we obtain

(4) $$\begin{array}{c} L^{\left(n-1\right)}f=\int_{0}^{t}F\left(\xi ,x,g,f,\frac{\partial f}{\partial x},\frac{\partial^{2}f}{\partial x^{2}},\ldots \right)d\xi +c_{1}\left(x\right). \end{array}$$

Again, by integrating (4), we have

(5) L(n−2)f=∫0t∫0ξF(τ,x,g,f,∂f∂x,∂2f∂x2,…)dτdξ+c1(x)t    +c2(x),

since we know that multiple integral can be reduce to a single integral by using integral property. Hence, we can write (5) in the following form:

(6) L(n−2)f=∫0t(t−ξ)F(ξ,x,g,f,∂f∂x,∂2f∂x2,…)dξ+c1(x)t+c2(x).

If we continue this process of integration, we can get final form as follows:

(7) f(x,t)=∫0t(t−ξ)n−1(n−1)!F(t,x,g,f,∂f∂x,∂2f∂x2,…)dξ  +c1(x)tn−1(n−1)!+c2(x)tn−2(n−2)!+⋯cn(x).

By writing the constant of integration in the form *c*_*k*_(*x*) = (∂*f* ^*n*−*k*^(*x*, 0^+^))/∂*t* ^*n*−*k*^, *k* = 1,…, *n* and substituting (2) in (7) then (7), we have

(8) f(x,t)=∑k=0n−1∂kf(x,0+)∂tktkk! +∫0t(t−ξ)n−1(n−1)!(Rf+Nf+g)dξ.

In iteration form (8), it can be written as follows:

(9) fj+1(x,t)=f0(x,t)+∫0t(t−ξ)n−1(n−1)!(Rfj+Nfj+g)dξ,                       j=0,1,2,…,

where *f*_0_(*x*, *t*) = ∑_*k*=0_^*n*−1^((∂^*k*^ *f*(*x*, 0^+^))/∂*t* ^*k*^)(*t* ^*k*^/*k*!).

In (9), (*t*−*ξ*)^*n*−1^/(*n* − 1)! is Lagrange multiplier of He's variational iteration method, denoted by *λ*, if *n* is an odd integer, and (9) can be written in standard variational iteration algorithm II [3]

(10) $$\begin{array}{c} f_{j+1}\left(x,t\right)=f_{0}\left(x,t\right)+\int_{0}^{t}\lambda \left(Rf_{j}+Nf_{j}+g\right)d\xi ,\\ f_{0}\left(x,t\right)=\sum_{k=0}^{n-1}\frac{\partial^{k}f\left(x,0^{+}\right)}{\partial t^{k}}\frac{t^{k}}{k!},\;\;\lambda =\frac{{\left(t-\xi \right)}^{n-1}}{\left(n-1\right)!}. \end{array}$$

Equation (10) is exactly the same as the standard He's variational iteration algorithm II [3]. Here is a point to be noted, if we change our initial guess by adding source term in it, the resulting formulation will give the results obtained by well-known Adomian decomposition method by decomposing the nonlinear term in (10). Consider

(11) $$\begin{array}{c} f_{j+1}\left(x,t\right)=\int_{0}^{t}\lambda \left(Rf_{j}+Nf_{j}\right)d\xi ,\\ f_{0}\left(x,t\right)=H\left(x,t\right),\;\;\lambda =\frac{{\left(t-\xi \right)}^{n-1}}{\left(n-1\right)!},\\ H\left(x,t\right)=\sum_{k=0}^{n-1}\frac{\partial f\left(x,0^{+}\right)}{\partial t^{k}}\frac{t^{k}}{k!}+\int_{0}^{t}\lambda g\left(x,\xi \right)d\xi . \end{array}$$

Equation (11) is an alternative approach of Adomian decomposition method, where *H*(*x*, *t*) is a term which arises from prescribed initial condition and source term. Furthermore, if we decompose the term *H*(*x*, *t*) in (11) and write the resulting equation in the form

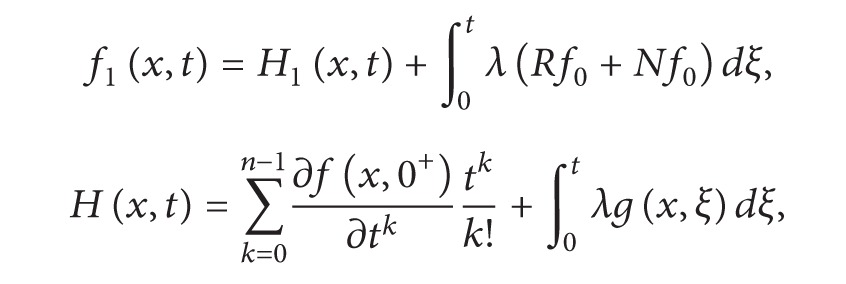
(12)

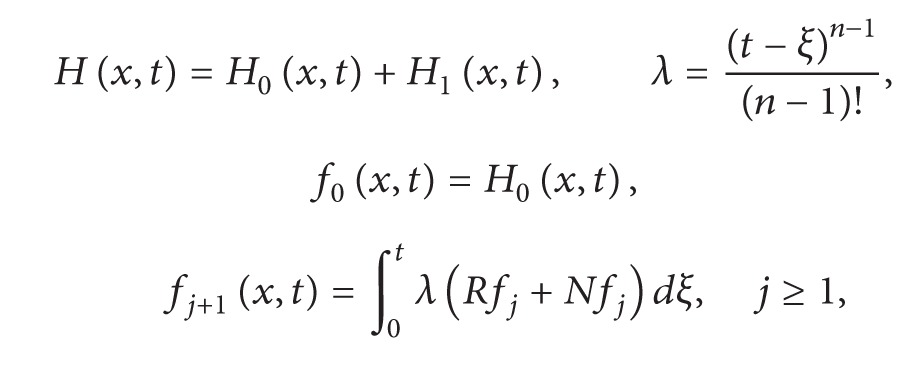
(13)
equation (2) is an alternative form of modified Adomian decomposition method.

## 3. Illustrative Examples

In order to illustrate the solution procedure, we consider the following examples for ordinary and partial differential equations.

**Example 1**

Consider the Blasius equation

(14) $$\begin{array}{c} u^{\prime \prime \prime }\left(x\right)+\frac{1}{2}u\left(x\right)u^{\prime \prime }\left(x\right)=0, \end{array}$$

subject to the boundary conditions

(15) $$\begin{array}{c} u\left(0\right)=0,\;\;u^{\prime }\left(0\right)=1,\;u^{\prime }⟶0,\;\;x⟶\infty . \end{array}$$

To solve the above given problem, we consider an extra initial condition; that is, *u*′′(0) = *α*. In order to solve (14) with this extra initial condition, we follow the formulation given in (10). Consider

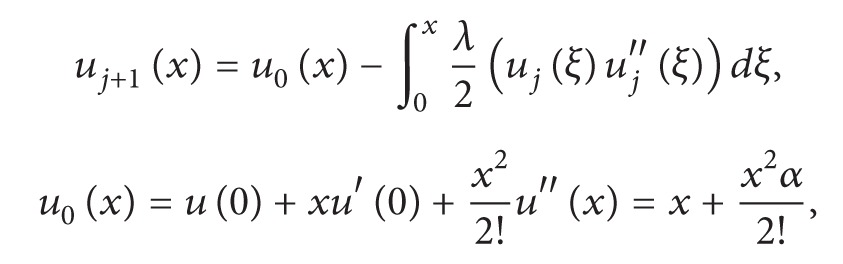
(16)

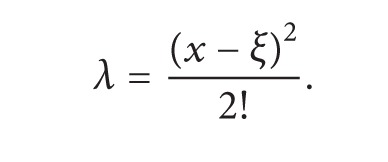
(17)
By using (16), we obtain the following successive approximations:

(18) u1(x)=x+αx22−αx448−α2x5240,u2(x)=x+αx22−αx448−α2x5240+αx6960+11α2x720160 +11α3x8161280−α2x9193536−α3x10518400−α4x115702400,⋮

Equation (18) is the exactly the same as obtained by using classical VIM in [20] and one can find the value of *α* by using Padé approximant [21].

**Example 2**

Consider the nonhomogeneous wave equation

(19) $$\begin{array}{c} \frac{\partial^{2}u\left(x,t\right)}{\partial t^{2}}=\frac{\partial^{2}u\left(x,t\right)}{\partial x^{2}}+\eta \left(x,t\right), \end{array}$$

where *η*(*x*, *t*) = 2*e* ^−*πt*^sin*πx*, subject to the initial conditions

(20) $$\begin{array}{c} u\left(x,0\right)=\sin\pi x,\;\;u_{t}\left(x,0\right)=-\pi \sin\pi x, \end{array}$$

whose exact solution is

(21) $$\begin{array}{c} u\left(x,t\right)=e^{-\pi t}\sin\pi x. \end{array}$$

To solve (19), we follow the formulation, given in (11). Consider

(22) uj+1(x,t)=∫0tλ(∂2uj∂x2)dξ,u0(x,t)=H(x,t),  λ=(t−ξ),H(x,t)=sinπx−tπsinπx+∫0t(t−ξ)(2π2e−πξsinπx)dξ,u0(x,t)=H(x,t)=−sinπx+tπsinπx +2e−πtsinπxuj+1(x,t)=∫0t(t−ξ)(∂2uj∂x2)dξ,u1(x,t)=(2−2πt+π2t22!−π3t33!)sinπx−2e−πtsinπx,u2(x,t)=(−2+2πt−π2t2+π3t33−π4t44!+π5t55!)sinπx −2e−πtsinπx,u3(x,t)=(2−2πt+π2t2−π3t33+π4t43(4) −π5t53(4)(5)+π6t66!−π7t77!)sinπx −2e−πtsinπx,⋮

Upon summing these iterations, we observe that

(23) u(x,t)=(1−πt+π2t22!−π3t33!+π4t44!−π5t55! +π6t66!−π7t77!+⋯)sinπx≈e−πtsinπx.

Solution (23) is exactly the same as obtained by using ADM in [22].

## 4. Conclusion

This paper helps us to gain insight into the idea of Adomian decomposition method and variational iteration method. By keeping in view both methods, we propose more simplified forms to calculate Lagrange multipliers. By introducing this Lagrange multiplier in ADM and VIM following the observations that have been made,

- there is no need to do integration process again and again like we do in Adomian decomposition method and one can get the same results of Adomian method.
- It is easy to calculate the Lagrange multiplier of He's variational iteration method.
- This new approach avoids the unnecessary calculations like we did in He's variational iteration method and Adomian decomposition method.
- This study shows that VIM is another form of expressing ADM and vice versa.

So we can say that the present method is parallel form of ADM and can give good results of VIM with less effort.

## References

[1] Kevorkian J, Cole JD (1996). *Multiple Scale and Singular Perturbation Methods*.

[2] He J-H (1999). Variational iteration method—a kind of non-linear analytical technique: some examples. *International Journal of Non-Linear Mechanics*.

[3] He JH, Wu GC, Austin F (2009). The variational iteration method which should be followed. *Nonlinear Science Letters A*.

[4] Faraz N, Khan Y, Austin F (2010). An alternative approach to differential-difference equations using the variational iteration method. *Zeitschrift fur Naturforschung A*.

[5] Khan Y (2009). An effective modification of the laplace decomposition method for nonlinear equations. *International Journal of Nonlinear Sciences and Numerical Simulation*.

[6] Khan Y, Faraz N (2011). Application of modified Laplace decomposition method for solving boundary layer equation. *Journal of King Saud University*.

[7] Khan Y, Austin F (2010). Application of the laplace decomposition Method to Nonlinear Homogeneous and Non-Homogenous Advection Equations. *Zeitschrift fur Naturforschung A*.

[8] Khan Y, Latifizadeh H (2014). Application of new optimal homotopy perturbation method and Adomian decomposition methods to MHD non-Newtonian fluid flow over a stretching sheet. *International Journal of Numerical Methods for Heat and Fluid Flow*.

[9] Chun C, Jafari H, Kim Y-I (2009). Numerical method for the wave and nonlinear diffusion equations with the homotopy perturbation method. *Computers and Mathematics with Applications*.

[10] Khan Y, Wu Q, Faraz N, Yildirim A (2011). The effects of variable viscosity and thermal conductivity on a thin film flow over a shrinking/stretching sheet. *Computers and Mathematics with Applications*.

[11] Khan Y, Wu Q (2011). Homotopy perturbation transform method for nonlinear equations using He’s polynomials. *Computers and Mathematics with Applications*.

[12] Adomian G (1994). *Solving Frontier Problems of Physics: The Decomposition Method*.

[13] Rach R (1987). On the Adomian (decomposition) method and comparisons with Picard’s method. *Journal of Mathematical Analysis and Applications*.

[14] Wazwaz A-M (2000). The decomposition method applied to systems of partial differential equations and to the reaction-diffusion Brusselator model. *Applied Mathematics and Computation*.

[15] Jafari H, Daftar-Geijji V (2007). Revised Adomian decomposition method for solving a system of nonlinear equations. *Applied Mathematics and Computation*.

[16] Rach RC (2008). A new definition of the Adomian polynomials. *Kybernetes*.

[17] Inokuti M, Sekine H, Mura T, Nemat-Naseer S (1978). General use of the Lagrange multiplier in nonlinear mathematical physics. *Variational Method in the Mechanics of Solids*.

[18] Xu L, He J-H, Wazwaz A-M (2007). Variational iteration method-Reality, potential, and challenges. *Journal of Computational and Applied Mathematics*.

[19] He J-H (2007). Variational iteration method-Some recent results and new interpretations. *Journal of Computational and Applied Mathematics*.

[20] Wazwaz A-M (2007). The variational iteration method for solving linear and nonlinear systems of PDEs. *Computers and Mathematics with Applications*.

[21] Wazwaz A-M (2007). The variational iteration method for solving two forms of Blasius equation on a half-infinite domain. *Applied Mathematics and Computation*.

[22] El-Gamel M (2007). Comparison of the solutions obtained by Adomian decomposition and wavelet-Galerkin methods of boundary-value problems. *Applied Mathematics and Computation*.

[23] Jafari H, Tajadodi H, Baleanu D (2013). A modified variational iteration method for solving fractional Riccati differential equation by Adomian polynomials. *Fractional Calculus and Applied Analysis*.

